# Development of quality indicators for monitoring outcomes of frail elderly hospitalised in acute care health settings: Study Protocol

**DOI:** 10.1186/1472-6963-11-281

**Published:** 2011-10-20

**Authors:** Caroline A Brand, Melinda Martin-Khan, Olivia Wright, Richard N Jones, John N Morris, Catherine M Travers, Joannne Tropea, Leonard C Gray

**Affiliations:** 1 Department of Clinical Epidemiology, Biostatistics and Health Services Research, Melbourne Health, The University of Melbourne and The Royal Melbourne Hospital, Melbourne, Australia; 2 Department of Medicine, University of Melbourne, Parkville, Victoria, Australia; 3 Centre for Research Excellence in Patient Safety (CREPS) Monash University, Melbourne, Victoria, Australia; 4 Centre for Research in Geriatric Medicine, The University of Queensland School of Medicine, Woolloongabba, Queensland, Australia; 5 Nutrition and Dietetics, School of Human Movement Studies, The University of Queensland, St Lucia, Australia; 6 Harvard Medical School, Cambridge, Massachusetts, USA; 7 Institute of Ageing Research, Boston, Massachusetts, USA

## Abstract

**Background:**

Frail older people admitted to acute care hospitals are at risk of a range of adverse outcomes, including geriatric syndromes, although targeted care strategies can improve health outcomes for these patients. It is therefore important to assess inter-hospital variation in performance in order to plan and resource improvement programs.

Clinical quality outcome indicators provide a mechanism for identifying variation in performance over time and between hospitals, however to date there has been no routine use of such indicators in acute care settings.

A barrier to using quality indicators is lack of access to routinely collected clinical data. The interRAI Acute Care (AC) assessment system supports comprehensive geriatric assessment of older people within routine daily practice in hospital and includes process and outcome data pertaining to geriatric syndromes.

This paper reports the study protocol for the development of aged care quality indicators for acute care hospitals.

**Methods/Design:**

The study will be conducted in three phases:

1. Development of a preliminary inclusive set of quality indicators set based on a literature review and expert panel consultation,

2. A prospective field study including recruitment of 480 patients aged 70 years or older across 9 Australian hospitals. Each patient will be assessed on admission and discharge using the interRAI AC, and will undergo daily monitoring to observe outcomes. Medical records will be independently audited, and

3. Analysis and compilation of a definitive quality indicator set, including two anonymous voting rounds for quality indicator inclusion by the expert panel.

**Discussion:**

The approach to quality indicators proposed in this protocol has four distinct advantages over previous efforts: the quality indicators focus on outcomes; they can be collected as part of a routinely applied clinical information and decision support system; the clinical data will be robust and will contribute to better understanding variations in hospital care of older patients; The quality indicators will have international relevance as they will be built on the interRAI assessment instrument, an internationally recognised clinical system.

## Background

Admissions to acute hospitals of very old patients are common [1] and, in some settings, are increasing at a far greater rate than other age groups [2].

Frail older people in acute care hospitals are at risk of a range of adverse outcomes (including geriatric syndromes) in association with the hospital episode, including functional loss, delirium, loss of morale, falls, pressure ulcers, poly-pharmacy, prolonged hospital stay, discharge to nursing home care and early readmission [3-5]. A range of associated socio-economic costs include increased length of hospital stay and expenditure, higher mortality, persistent decline after discharge including loss of ability to perform activities of daily living, recurrence of illness leading to readmission, and placement in permanent residential care [3].

The quality of care provided to the frail aged in acute hospitals influences patient safety, health outcomes, mortality, discharge destination and the likelihood of hospital readmission [6-8]. Good care and management strategies appear to improve clinical outcomes for these patients, particularly if carefully targeted [9]. There is also evidence that some strategies directed at prevention or treatment of individual geriatric syndromes (e.g. delirium, functional decline) may be effective [10-12]. In this context, one aspect of assessing a hospital's performance might be to ask how well these problems (or potential problems) are identified, prevented and managed. It is likely that hospitals and treating units vary in their performance in implementing strategies to impact positively on these outcomes.

Quality outcome and process of care quality indicators (QI) provide a mechanism to identify variation in performance and can contribute to a framework for improving performance. At present, indicators of hospital quality in the health system include: achieving accreditation, waiting times for elective surgery and cost per casemix-adjusted separation. These are hospital-centred outcomes rather than patient-centred outcomes, and do not provide a measure of the quality of care provided to individual patients, or patient outcomes [13]. More recent health care reform policy indicates increasing interest in measuring quality of care outcomes [14,15]. However, to our knowledge there is no routine use of outcome-based QIs for monitoring the quality of acute care for the illnesses and syndromes characteristic of old age despite the large emphasis on quality of care for these patients in the literature over the past 10 years [16].

A purpose built set of geriatric specific *process *quality indicators, Acute Care of the Vulnerable Elders-3 (ACOVE-3), has been developed in the United States (US) for community-dwelling vulnerable elders (VEs). These indicators are applied to older persons who are assessed as VEs using the vulnerable elders survey [17], which is predictive of patients at higher risk of functional decline and death [18]. A subset of these QIs is applicable to care in the acute hospital setting and the VE survey can be applied at admission to hospital [17]. The ACOVE-3 process indicators are a comprehensive set of QIs specifically designed for older people that relate to the acute care setting. They were developed by researchers at the University of California and the Research and Development Corporation (RAND), in response to analyses of national data sets that demonstrated deficits in the quality of care of older people [16,19,20]. Extensive literature reviews of evidence-based practice informed ACOVE's development and face validity was assessed by multidisciplinary panels of clinical experts [16,21]. Versions 1 (1999, no age specified), 2 (2001, for those 75+ years) and 3 (2006, for those 65+ years) of the ACOVE indicators are obtained from medical file reviews and focus on care inputs and processes rather than care outcomes. Since publication of the ACOVE QIs, additional aged care QIs have been published, however these are also primarily process QI [22] and/or are focused on specific aspects of aged care management such as cognitive impairment [23,24].

Process indicators are generally considered to be easier to measure due to a general lack of outcome data documented in patient medical records, a problem acknowledged in the peer-reviewed literature [25]. However, they do not facilitate the measurement of patient outcomes and can only measure quality of care at a single point in time, not over a period of time.

Routinely collected data, for instance within electronic patients records, clinical registries and administrative data systems offers another source of potential outcome QIs. In the US, the Agency for Healthcare Research and Quality (AHRQ) collects QIs based on hospital administrative datasets in the US and include prevention QIs; inpatient QIs; patient safety indicators and paediatric QIs [26]. These are not specifically designed for older acute inpatients and include few indicators (for example, in-hospital fracture and decubitus ulcer) pertaining to geriatric syndromes. The AHRQ QIs have been translated into 18 Australian patient safety indicators, for application with the ICD-10 Australian Modification coding system, however comprehensive implementation and evaluation has not yet been performed [27].

Another potential geriatric specific data source for outcome quality QIs is the interRAI AC assessment system. The interRAI AC is designed to support comprehensive geriatric assessment of older patients in the acute hospital setting [28-30]. It includes data pertaining to geriatric syndromes (and risks of acquiring them) and can be utilised to collect patient outcome data, as it includes data collection points in the pre-morbid, admission and discharge periods. It is designed for the comprehensive assessment of frail older adults in acute hospitals and consists of a broad schedule of items designed to characterize the patient's medical, functional and psycho-social characteristics and contains outcome measures that are collected at discharge from hospital. It provides comprehensive information about the person's status in the pre-morbid period, as well as at admission and discharge and has the potential to provide a foundation for QIs.

The interRAI AC is a purpose built "3^rd ^generation assessment tool" and is part of an integrated multi-domain suite of assessment instruments [29]. Data is entered into a web-based system that generates reports and triggers in real time. In this sense it is without peer in acute care. In a recently completed large multi-centre study of this instrument in 9 countries, high levels of inter-rater reliability were reported with weighted kappa scores averaging > 0.6 for pre-morbid and admission items [30]. These scores were consistent across items that require considerable clinical judgement (e.g. ADL and cognition) or those that involve information reported from carers. Application of the interRAI AC results in the identification and documentation of a comprehensive range of geriatric syndromes and risks, in contrast to the current paucity of documentation of these parameters in patient medical files noted in the literature [25]. It has the distinct advantage over other assessment systems of being part of a comprehensive suite of multi-domain instruments that permits sharing and transfer of comparable information across care settings.

The interRAI research collaborative has an extensive history of successful QI development, primarily in long term institutional and community care [31,32]. A set of outcome-based QIs were developed for long-term care settings in the US through comprehensive literature reviews, clinical panels and the comparison of facility performance and risk adjustment using a standardized dataset obtained with the interRAI Home Care (HC) and interRAI Nursing Home Care instruments [33].

This paper reports the protocol for the development of outcome oriented QIs that was successfully presented to the Australian National Health and Medical Research Council and received funding in 2009.

The primary aims of the project are to develop, using the interRAI AC, outcome oriented QIs in relation to common geriatric syndromes and functioning for the care of the frail aged in acute care.

Secondary aims of the project are to:

• explore the relationship of existing ACOVE process QIs with clinical outcomes and outcome oriented QIs developed in the project,

• explore the relationship of potential QIs that can be derived from existing administrative datasets with outcome oriented QIs developed in this project, and

• compare the cost of each method of QI derivation and determine which provides the most effective and reliable set of QIs.

## Methods/Design

### Rationale for use of the interRAI AC for the development of QIs

The rationale for selecting the interRAI AC clinical data set as a starting point for the development of QIs is as follows:

■ The instrument is designed primarily to support clinical decision making. If it is used for this purpose, QIs would be available without additional effort from clinicians or coders,

■ The item set has been in development for over 10 years with extensive input from many international clinicians and scientists. It is in its second version, and may become widely used around the world,

■ The majority of individual items have been thoroughly tested, have good inter-reater reliability (> 0.6) and are mature and stable. They have recently been re-tested in a multi-national study,

■ The data is collected at 3 time points (pre-morbid/admission/discharge) providing an unique opportunity to monitor outcomes,

■ There is a growing cadre of clinicians experienced in using and interpreting the instrument, and

■ There are no similar, second or third generation assessment tools available internationally designed for hospital use.

In addition there are some logistic reasons to use this data set:

■ The Centre for Research in Geriatric Medicine, Queensland, Australia has over 5 years experience in using the instrument,

■ There is a formal training program available for nurse assessors, and

■ Web-based software is available for data collection.

Further, by using the interRAI AC platform and collaborating with international investigators at Harvard University, Boston, US (RJ, JM), there is a vastly greater opportunity for QIs developed by an Australian team to be used internationally.

### Preliminary Data

The design of the study has been informed by a preliminary study on geriatric outcomes in an acute care hospital that was conducted at Princess Alexandra Hospital in Brisbane in 2006-7 using the interRAI AC. Information was derived from preliminary QIs, designed to provide estimates of incidence using the interRAI AC data set. This data was collected as part of a larger study designed to develop a screening tool to determine which patients would benefit from comprehensive geriatric assessment within a general medical population. The study secured a consent rate of approximately 90% and, at the time of writing, data for 202 participants aged between 70 and 102 years (median 80 years; 51% males) was available for analysis. Candidate QIs which can be derived from the interRAI AC are shown below (Table 1), together with their observed incidence in medical patients > 70 years with an anticipated length of stay of at least 48 hours. The functional decline QIs compare data on the patient's functioning at discharge compared to that obtained for the pre-morbid period.

**Table 1:** Incidence of candidate QIs from the interRAI AC in a sample of 202 elderly hospital inpatients

Candidate QIs from the interRAI AC	Incidence (%)
Fall during hospitalisation	8
Pressure ulcer (new or worsening)	8
Delirium during hospitalisation	19
Functional decline (premorbid to discharge)	
Decline in ability to communicate	2
Decline in cognitive function	5
Decline in Activities of Daily Living (ADL) function	16
Decline in Instrumental ADL (IADL) function	41
Decline in bladder or bowel continence	10
Discharge to a higher level care	22
Readmission within 28 days	14
Death in hospital	4

While these QIs are tentative, they provide an indication of the incidence of these adverse outcomes in hospital that was instructive in formulating the study proposal.

Other preliminary studies conducted by authors LG and OW have shown significant proportions of under-documentation of geriatric syndromes and risks in patient medical files (unpublished data). A review of 100 charts of general medical patients aged 70 years or more, showed between 80%- 90% of medical files did not contain any documentation about the patients' cognitive skills for daily decision making; periodic disordered thinking/awareness; short term memory recall; comprehension; behaviour; eating ability; balance (transitions); use of urinary collection device and falls. These rates of under-documentation are consistent with the peer-reviewed literature [25]. More importantly, an analysis of the ACOVE-3 QIs in the acute care setting (n = 328), showed rates of adherence for items grouped as geriatric QIs (delirium, dementia, pressure ulcers, physical function), to be significantly lower than the general medical QIs respectively (81.5%, [95% CI 79.3-83.7%] vs. 61.6%, [95% CI 59.1 - 64.1]) (p < 0.01) [18]. This was explained by discrepancies in screening, diagnosis and therapy [18].

The study will be conducted in 3 phases:

1. Development of a preliminary QI set,

2. Field study, and

3. Analysis and compilation of a definitive QI set.

### Phase 1: Development of candidate set of QIs derived from the interRAI AC instrument

This phase will involve preparatory work, including review of the relevant scientific literature pertaining to adverse geriatric outcomes in the acute hospital, culminating in a 2-day workshop with a panel of clinical experts. This work will involve the conceptualisation of a set of QIs for care of older adults in acute care hospitals. A panel of 10 geriatricians including representatives of the Australian and New Zealand Society for Geriatric Medicine, general physicians, nurses and allied health experts will be invited to the workshop to review (and refine) the indicators available. Once conceptual models are devised, the indicators will be defined precisely in interRAI terms, including how they will be measured. The style and format of the QIs will be discussed and determined. These could include "failure to improve" and "preventable decline" indicators as well as QIs reflecting sentinal events such as falls and pressure ulcers. Following definition of the interRAI AC indicators, the experts will select a set of relevant ACOVE-3 QIs to apply in the field studies. Process indicators selected from ACOVE-3 will be matched with outcome data available from the interRAI AC to facilitate a comparison of care processes and outcomes. Criteria for the selection of ACOVE-3 indicators will be ease of measurement from chart review [18]. A pathway for training research staff to locate the chosen ACOVE-3 measures by file review will be developed. Data that might potentially be sourced from AHRQ administrative data sets (US) and similar Australian hospital administrative datasets will also be defined.

### Phase 2: Field study

Phase 2 will be a prospective study of the QIs selected in phase 1 in a representative sample of older persons admitted to acute care hospitals.

#### Subjects and recruitment procedure

For the collection of interRAI AC data, all patients who are aged 70 years and older, admitted to general medical wards and who are likely to stay in hospital for at least 48 hours will be invited at admission to participate in the study. Personal or proxy consent will be obtained in writing prior to commencement of the study. Recruitment will be restricted to weekdays, because of the difficulties in recruiting suitable staff on weekends. Discharge assessments will be completed on the day of discharge. In the case of weekends, in our experience, discharges can be identified on Fridays, and provisional data collected in advance, with final verification on Monday morning.

#### Data Collection

An assessment schedule will be administered within 24 hours of admission to the ward for consenting patients. Patients or their proxies will complete a Vulnerable Elders Survey-13 (VES-13). Patients obtaining scores of 3 or more on the VES-13, or proxies will be questioned at greater depth in an interview about functional status, ADLs and IADLs to establish risk of functional decline. ADL and IADL items from the Medicare Current Beneficiary Survey will be used to maintain consistency with the VES-13 development literature [17]. This procedure is proposed to enable direct comparison of our data with studies undertaken in the US [18]. All patients (both those identified and not identified as VEs) will have a full interRAI AC assessment completed by a trained nurse assessor. The interRAI AC instrument will be utilized to characterize the general status of the subjects and will collect the outcome QIs selected during phase 1. Conducting this assessment on all consenting patients will facilitate assessment of the ability of the VES-13 to predict patient outcomes and the specific QIs.

The interRAI AC assessment focuses on the first 24 hours of admission to the ward as well as premorbid and demographic information. This 24-hour timeframe for the admission assessment enables an adequate period of observation to characterise key parameters including behaviour and functional ability. The "baseline pre-morbid period" is defined as the three days prior to the onset of the illness precipitating the admission.

The trained nurse assessor will monitor patients every day of the admission to accurately record the incidence of adverse outcomes, e.g. falls or delirium, and to ensure adequate notice of impending discharge. At discharge, a full assessment of the subject's status will be conducted. Administrative data on 28 day readmission and post-discharge change of living arrangement will be collected for all patients through a follow-up telephone call to the patient's home or carer by the nurse assessor. This is to determine if the participant has been subsequently admitted to any acute care hospital, residential care, and whether they were still alive. Verification of admission will be sought from the relevant institution, in instances of readmissions. Medical file reviews on all participating patients will be conducted using the selected set of ACOVE-3 QIs following the patient's discharge. A file examination will be conducted to compare the levels of documentation of the selected ACOVE-3 QIs with the information collected from the interRAI AC assessments conducted in phase 1. Evidence of documentation will be graded based on the available methodology already developed for measuring the ACOVE-3 QIs in patient files [18].

All data in this study will be collected by nurse assessors with experience in aged care. Commencing and concluding times for the assessments will be documented. There will be one nurse, full-time, in each hospital to complete the VES-13 and the interRAI AC assessments, daily monitoring of adverse events and follow-up telephone calls. To avoid potential bias, the nurse completing the VES-13 and the interRAI AC will not conduct the ACOVE-3 file reviews. At all sites, a nurse or trained research assistant experienced with reading medical files will undertake the ACOVE-3 file review. This will ensure that the person conducting the file reviews is blinded to any preceding information about patients. The ACOVE-3 reviewer will be blinded to the interRAI AC data. Formal training programs will be conducted to ensure high quality performance in administration of the assessment instruments (three days for interRAI AC; one day for ACOVE-3). File reviewers will undergo three days training and inter-rater reliability for the ACOVE-3 file review will be calculated on 10 cases collected in the run-in period.

The Centre for Research in Geriatric Medicine has extensive experience in training assessors for these procedures.

#### Study settings

Ten hospitals initially provided in principle agreement to participate in the study and nine formally agreed and are participating. These include five metropolitan tertiary teaching hospitals and four regional hospitals. This array of hospitals is designed to reflect the hospital environment to which frail older people are most commonly admitted. This is balanced against the need for proximity to supervise the research, conduct training and to access essential specialist geriatrician or psycho-geriatrician expertise. Therefore remote settings have not been included.

### Phase 3: Analysis and final consultation (with expert panels)

This phase will comprise the latter stages of analysis of results of the field study, preparation of reports to inform the expert panel, a two day seminar to consider the findings of the field study, and assembly of the final QI set and associated recommendations. A formal report will be prepared for general scrutiny in addition to publications for the peer-reviewed literature. A formal procedure for the selection of the final QI set will be incorporated into the expert panel deliberations, similar to that used in assembly of the ACOVE-3 indicators. This process involves two rounds of anonymous ratings on a risk-benefit scale with group discussion between rounds [22,34].

### Statistical Analysis

The sample will include 480 cases and the adequacy of that sample size was assessed using two simulation methods. The planned sample size will have 85% power to detect reliability coefficients within an acceptable level of precision (estimated coefficient > 0.4 when the true value is 0.6). For the logistic regression models, setting power to correctly identify poor focal units (individual hospital) with the QI score at 80%, we will be able to correctly classify units as poor if their true odds ratio (OR) of having more clients flagging the QI definition is at least 1.4 times the OR in the reference sample (for reasonable ranges of QI scores in the reference sample: see Table 2). This magnitude of effect size (> 0.19) defines the lower bounds of small effects [35,36]. Smaller samples would result in less stable estimates of reliability and validity.

**Table 2:** Development of sample size estimations using simulation methods

	QI Score for focal Unit		Odds Ratio for focal unit	
Reference sample QI score	80% hit*	95% hit	80% hit	95% hit
0.05	0.09	0.16	1.88	3.62
0.10	0.16	0.25	1.71	3.00
0.20	0.25	0.32	1.33	1.88
0.30	0.37	0.44	1.37	1.83
0.40	0.48	0.57	1.38	1.99
0.50	0.57	0.65	1.33	1.86

* Hits are defined as the correct identification of facilities providing poorer quality of care as defined by a higher QI score.

The collected data will be primarily analysed to evaluate the appropriate casemix, risk adjustment and reliability of the new QIs coded from the interRAI AC assessment system, and to compare them with the ACOVE QIs, as outlined in the study hypotheses. The QIs will be adjusted for ascertainment and selection bias through risk adjustment procedures [37]. The determination of appropriate casemix and risk adjustment procedures will involve simple bi-variable descriptive statistics (correlations, mean differences). Good candidates for adjustment will be included as matching criteria in the QI adjustment process. The QI adjustment method will use a procedure that has the advantage of being quasi-parametric, involving matching individual patients in target facilities to randomly selected patients from other facilities. This counterfactual contrast will include a re-sampling procedure and allows QIs to be expressed as odds ratios or expected proportions given an overall average rate and an empirically based replication interval (confidence interval). Relative to extant methods of QI adjustment (e.g., US Centers for Medicare and Medicaid Services Nursing Home Quality Measures) this approach is relatively simple, can be implemented in settings or clinical sub-populations of very small size and represents as perfect as possible adjustment for differences in the patient mix across clinical settings.

The reliability of QI scores will be evaluated by multiple bootstrapped spit-half correlations of patient samples and time-to-time correlations of repeated QI scores. This is a unit-level analysis, where for each facility a bootstrapping data augmentation approach is used to generate 20 random half samples of clients. Comparisons with ACOVE QIs will use standard methods for comparing correlation coefficients for contrasting reliability coefficients, and cross-tabulations of tertiles of QIs in similar domains for the validity assessment. A small cost analysis will be conducted to compare the costs of the ACOVE-3 and interRAI AC methods. The major cost will be personnel time. All research staff will keep time logs (e.g. for four-month intervals) during the interRAI AC assessment period and the ACOVE-3 file review period.

### Intervention Period

The planned intervention period was two years, based on estimations of admission and consent rates in several other projects concerning similar hospital populations. Recruitment and data collection have taken longer than anticipated, however data collection will be completed in 2011 and results should be available early in 2012.

This research protocol has been approved by the Human Research Ethics Committees at The University of Queensland and all participating hospitals.

## Discussion

There is a growing interest in the measurement of quality to support accountability in the health care sector and to improve services [37]. With demographic changes, older patients will become an increasingly important clientele of hospitals. It is also likely that, as the baby boomer generation approaches old age, there will be growing demand for evidence of quality care. These QIs provide one mechanism to appraise performance, and can provide an objective framework upon which to base quality improvement strategies.

The regular measurement of outcome-based QIs associated with common geriatric syndromes/risks in hospital is important, as it monitors: (i) the identification of important issues for older people in hospital and (ii) the efficacy of care provided to frail older patients in hospital based on individual outcomes. Assessment of hospital performance according to risk-adjusted QIs may form the basis of accreditation assessment and licensing processes. QIs which reflect the quality of care in relation to geriatric syndromes/risks would assist hospitals, and clinical service units within them, to appraise their performance, and to compare with other similar hospitals. Furthermore, they would assist in estimating the cost of different modes of care delivery at different hospital sites in relation to the patient outcomes achieved. In the US, the QIs contribute to assessing whether legislative requirements for care are met [18]. A recent innovation is the requirement that QIs derived from the interRAI nursing home instrument for every Medicare/Medicaid subsidised nursing home in the US, be made available on the internet for the general public to view when selecting a care provider [37].

There remains a lack of recognised QIs that focus on common geriatric syndromes and function, which directly reflect patient outcomes within acute care settings. Whilst structural and process indicators provide indirect evidence of service performance and have the advantage of being relatively easy to collect, file review is still required which is time and resource intensive and therefore costly.

Outcome QIs are important measures of quality of care and are of interest to all healthcare stakeholders. However, a key barrier to implementing outcome QIs in many settings is lack of access to routinely collected clinical outcomes data. Existing data sources, such as incident reporting systems [38] and routinely collected hospital episode data [39] have well recognised limitations and do not provide a complete set of important outcomes, whilst documentation within medical records is often suboptimal.

Overall, the approach to QI development proposed in this protocol has four distinct advantages. Firstly, the QIs focus on outcomes. Secondly, they can be collected as part of a routinely applied clinical information and decision support system, thereby reducing the cost substantially. The clinical data collected is robust and can contribute to better understanding variations in hospital performance for older hospitalised people based on benchmarking activities [40]. Finally, the QIs will have international relevance as they will be built on an internationally recognised clinical system.

## List of Abbreviations

AC: Acute Care; ACHS: Australian Council on Healthcare Standards; ACOVE: Assessing care of Vulnerable Elders; AHRQ: Agency for Healthcare Research and Quality; IADL: Instrumental Activities of Daily Living; NHMRC: National Health and Medical Research Council; OR: Odds Ratio; QI: Quality Indicator; US: United States; VEs: Vulnerable Elders; VES: Vulnerable Elders Survey

## Competing interests

The authors declare that they have no competing interests. Authors Gray, Morris and Jones are Fellows of the interRAI research consortium, which is a not-for-profit organization registered in the United States. Fellows contribute to the interRAI effort on a purely voluntary basis.

## Authors' contributions

CB, MMK, OW, CT, JT contributed to the study design and writing of the protocol manuscript. RJ and JM contributed to study design, particularly in relation to statistical methods and writing of manuscript. LG was responsible for the study concept and overall conduct of the study, and contributed to study design and writing of manuscript. All authors have read and approved the final draft of the paper.

## Pre-publication history

The pre-publication history for this paper can be accessed here:

http://www.biomedcentral.com/1472-6963/11/281/prepub
